# Quantitative Prediction of Microsatellite Instability in Colorectal Cancer With Preoperative PET/CT-Based Radiomics

**DOI:** 10.3389/fonc.2021.702055

**Published:** 2021-07-22

**Authors:** Jiaru Li, Ziyi Yang, Bowen Xin, Yichao Hao, Lisheng Wang, Shaoli Song, Junyan Xu, Xiuying Wang

**Affiliations:** ^1^ School of Computer Science, The University of Sydney, Sydney, NSW, Australia; ^2^ Department of Nuclear Medicine, Fudan University Shanghai Cancer Center, Shanghai, China; ^3^ Department of Oncology, Shanghai Medical College, Fudan University, Shanghai, China; ^4^ Department of Automation, Shanghai Jiao Tong University, Shanghai, China

**Keywords:** ^18^F-FDG PET/CT, microsatellite status, colorectal cancer, radiomics, machine learning

## Abstract

**Objectives:**

Microsatellite instability (MSI) status is an important hallmark for prognosis prediction and treatment recommendation of colorectal cancer (CRC). To address issues due to the invasiveness of clinical preoperative evaluation of microsatellite status, we investigated the value of preoperative ^18^F-FDG PET/CT radiomics with machine learning for predicting the microsatellite status of colorectal cancer patients.

**Methods:**

A total of 173 patients that underwent ^18^F-FDG PET/CT scans before operations were retrospectively analyzed in this study. The microsatellite status for each patient was identified as microsatellite instability-high (MSI-H) or microsatellite stable (MSS), according to the test for mismatch repair gene proteins with immunohistochemical staining methods. There were 2,492 radiomic features in total extracted from ^18^F-FDG PET/CT imaging. Then, radiomic features were selected through multivariate random forest selection and univariate relevancy tests after handling the imbalanced dataset through the random under-sampling method. Based on the selected features, we constructed a BalancedBagging model based on Adaboost classifiers to identify the MSI status in patients with CRC. The model performance was evaluated by the area under the curve (AUC), sensitivity, specificity, and accuracy on the validation dataset.

**Results:**

The ensemble model was constructed based on two radiomic features and achieved an 82.8% AUC for predicting the MSI status of colorectal cancer patients. The sensitivity, specificity, and accuracy were 83.3, 76.3, and 76.8%, respectively. The significant correlation of the selected two radiomic features with multiple effective clinical features was identified (p < 0.05).

**Conclusion:**

^18^F-FDG PET/CT radiomics analysis with the machine learning model provided a quantitative, efficient, and non-invasive mechanism for identifying the microsatellite status of colorectal cancer patients, which optimized the treatment decision support.

## Introduction

Colorectal cancer (CRC) is one of the most common malignancies in China and ranks the fifth in terms of cancer mortality (1). Microsatellite instability (MSI) is an essential molecular hallmark of hereditary non-polyposis colorectal cancer (HNPCC) and Lynch syndrome (LS). It also occurs in 15% of sporadic colorectal cancers and is often associated with the deficiency of the mismatch repair (MMR) system caused by the failure of one of the primary MMR genes, including MSH2, MLH1, MSH6, or PMS2 (2). CRCs with high MSI (MSI-H) are usually located in the right colon, more common in stage II, and relatively infrequent among metastatic tumors (3).

MSI is an essential factor in predicting the prognostic response and patient outcome. Retrospective studies demonstrated that patients with MSI-H CRCs had a better prognosis than those with stable microsatellite (MSI-L or MSS) tumors (4, 5), particularly in the cases of locally advanced stage II and stage III CRCs. However, the patients with MSI-H CRCs had a poor prognosis for stage IV CRCs, which constituted about 2–4% of all metastatic CRCs.

MSI contributed to the selection of the treatment strategy in CRC patients. For the chemotherapy, some randomized controlled trials showed no benefits from fluorouracil-based adjuvant chemotherapy in the patients with MSI-H CRCs, while adjuvant chemotherapy improved overall survival in patients with MSI-L or MSS tumors (6–8). Therefore, patients with stage II CRCs were recommended not to receive adjuvant chemotherapy in MSI status. Besides, MSI status also played a significant role in the selection of immunotherapy since MSI CRCs revealed highly upregulated expression of multiple immune checkpoints, including programmed death-1 (PD-1), programmed death-ligand 1 (PD-L1), and cytotoxic T lymphocyte-associated antigen 4 (CTLA-4), which caused tumors infiltrated by immune cells, primarily CD8+ tumor-infiltrating lymphocytes (TILs), T helper 1 (Th1) CD4+ TILs, and macrophages. Based on the principle involving the blockade of the immunoregulatory mechanisms, a trial of anti-PD-1 therapy called pembrolizumab showed that the immune-related objective response rate and immune-related progression-free survival rate were 40 and 78%, respectively, for mismatch repair-deficient (dMMR) CRCs (9). This trial was granted approval by the U.S. Food and Drug Administration (FDA) for patients with unresected or metastatic, MSI-H or dMMR solid tumors, including CRCs, in May 2017 (10).

Since MSI serves as a marker for predicting patient prognosis and responding to chemotherapy and immunotherapy, the identification of MSI status is critical for CRC patients. So far, various forms of testing methods have been applied in screening tumors for MSI or dMMR, including polymerase chain reaction (PCR) testing, immunohistochemical staining (IHC), and next-generation sequencing (NGS) (11). Testing for five DNA sequences by PCR and screening for loss of four MMR proteins (MLH1, PMS2, MSH2, and MSH6) expression by IHC are two standard reference methods recommended for detecting the MSI in CRC, both of which are complementary (12). MMR protein expression by IHC is performed to detect the absence or loss of a particular protein within the nucleus of the tumor cells, which can help identify the gene mutations resulting in truncation or increased degradation of the protein. However, in IHC, false negatives caused by missense mutation, and neoadjuvant chemoradiation in rectal cancer will reduce the staining intensity for MSH6. Moreover, compared with PCR testing, IHC results might be more affected by tissue fixation conditions. As for the PCR testing, it evaluates tumors by testing the repetitiveness of five DNA sequences, consisting of two mononucleotide loci (BAT25 and BAT26) and three dinucleotide loci (D2S123, D5S346, and D17S250). At least two unstable markers can define tumors as MSI-H. Compared with IHC, PCR requires normal tissue in addition to tumor tissue for comparison, and tumor microdissection, which makes the entire approach more expensive and complicated. Since those methods are all based on tumor tissues, the invasive biopsy is the only method for preoperative evaluation of MSI status for now. Therefore, the development of a non-invasive and objective method for assessing the MSI status will provide more diagnostic information for the precise treatment of CRCs.

Radiomics is considered a promising research field due to its potential to unveil disease characteristics through a non-invasive manner that fails to be appreciated by the naked eyes (13–15). It mines visual and subvisual quantitative imaging markers from high-throughput quantitative imaging features through machine learning and statistical modeling, followed by further quantitative analysis and analyzing the correlation with the clinical features. Furthermore, since radiomic features could reflect the underlying pathophysiology, and objectively and comprehensively evaluate the tumor heterogeneity, radiomics analysis has been applied within the clinical decision support system to improve the diagnostic, prognostic, and predictive accuracy (16).

Due to the non-invasive and low-cost properties of radiomics, it has been widely studied in various oncological fields with promising results. For example, radiomics has been reported to be able to predict the disease-free survival in early-stage lung cancer (17), prospectively measure the risk of breast cancer recurrence (18), and assess the treatment response after neoadjuvant therapy for rectal cancer (19). However, the prediction of MSI status in CRCs based on PET/CT-based radiomics is yet to be investigated. Thus, in this study, we aimed to investigate the efficiency of combining the preoperative ^18^F-FDG PET/CT radiomics with a machine learning model for predicting the microsatellite status of patients with colorectal cancer.

## Materials and Methods

### Patients Inclusion Criteria

We retrospectively investigated 262 patients with colorectal cancer in our hospital who underwent ^18^F-FDG PET/CT for initial staging before surgery, including 243 CRC patients obtained from January 2010 to July 2018 and 19 CRC patients recruited from February 2018 to March 2020. The surgery type (radical or palliative) was defined according to the tumor location and the stage of patients within 1 week after the PET/CT examination. Moreover, there were two exclusion criteria of the patients, namely, (1) the patients received neoadjuvant chemotherapy or radiotherapy due to clinically local advanced colorectal cancer and (2) the pathological results lacked IHC for MMR protein even though the patients received the operation. Therefore, for the patients within the 2010–2018 period, only 154 patients out of 243 patients diagnosed with colorectal cancer were enrolled in the present study, 141 of whom were MSS patients and 13 were MSI-H patients, while for the 2018–2020 period patients, they were all with stable microsatellite status and participated in the study. All patients were given written and informed consent for PET/CT procedures. This study was approved by the Ethics Committee of Fudan University Shanghai Cancer Center (No. 1909207-14-1910), and the data were anonymously analyzed.

### Assessment of MSI Status

The pathological results were all evaluated by professional pathologists, while the tumor stage was defined according to the fourth edition of the World Health Organization classification of tumors of the digestive system (20). The test for MMR gene proteins (MLH1, MSH2, MSH6, and PMS2) with IHC methods was used for the evaluation of the presence or absence of a functional MMR system (21). MSI-H CRCs were identified indirectly as tumors with loss of an MMR protein, while the tumors with intact MMR proteins could be considered as proficient MMR (pMMR) and were classified as MSS or MSI-low (MSI-L). If the result of testing was still in doubt, it was confirmed by the number of unstable microsatellite markers among five microsatellite markers within the PCR test. MSI-H status was identified if the number was above two.

### Medical Image Acquisition and Reconstruction Parameters

All the patients were fasted for 4–6 h before PET/CT to make blood glucose level under 11.1 mmol/L at the time of FDG injection. The examination was initiated 1 h after intravenous injection of ^18^F-FDG (7.4 MBq/kg (0.2 mCi/kg) of body weight). ^18^F-FDG PET/CT scanning was performed on a Siemens biograph 16HR PET/CT scanner (Knoxville, TN, USA), with 4.1 mm transaxial intrinsic spatial resolution (full width at half maximum) and 16.2 cm axial field width. Firstly, an unenhanced low-dose CT scan using a 120 kV automatic mA modulation range of 130–370 mA was acquired. Immediately after the CT scan, a PET scan was acquired in a three-dimensional mode. PET acquisition time was 3–4 min per bed position. The PET data were reconstructed iteratively by applying the CT data for attenuation correction, and the co-registered images were displayed on a workstation. The images were reviewed and manipulated in a multi-modality computer platform (Syngo, Siemens, Knoxville, TN, USA). Two experienced nuclear medicine physicians, unaware of clinical information, evaluated the images independently. The reviewers reached a consensus in cases of discrepancy. The region of interest was delineated on the primary tumor site. The maximum standardized uptake (SUVmax), mean standardized uptake (SUVmean), and metabolic volume (MTV) were measured from 3D isocontour at 40% of maximal pixel value, while total lesion glycolysis (TLG) was calculated by the multiplication among SUVmean and MTV.

### Medical Image Delineation

ITP-SNAP software (Version 3.6, USA) (22) was used for delineating the ROIs on PET images independently by two attending physicians from the department of nuclear medicine, who were blinded to the pathologic and MSI status results. The delineation was conducted on PET axial images layer by layer and corresponded to CT images based on the coordinate transformation and interpolation. Only the primary colorectal tumors were marked, and the ROIs were confirmed through discussion of two physicians when facing the controversy, especially for the tumors of which the contours were adjacent to the bladder and normal guts.

### PET/CT Radiomics Analysis With Machine Learning

**Figure 1:**
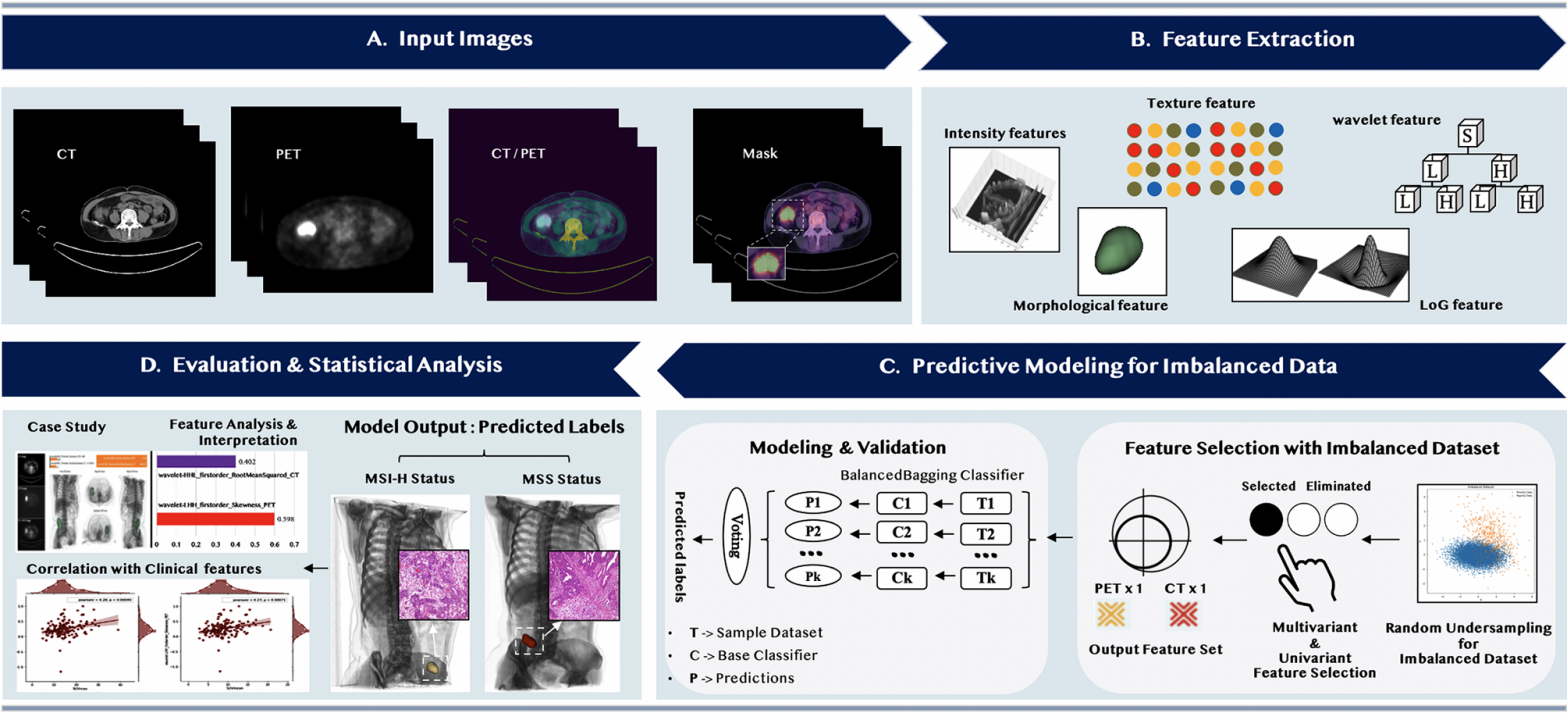
The overall flowchart of predicting the microsatellite instability status of the colorectal cancer patients.

Three principal stages of radiomics analysis were illustrated in **Figure 1**, namely, (1), feature extraction, (2) predictive modeling for imbalanced data, and (3) evaluation and statistical analysis. Firstly, after the manual delineation of the volumes of interest (VOIs) from PET images and mapping the VOIs to CT images, the feature extraction methods would be applied to automatically extract the quantitative imaging features from both PET and CT VOIs. The second stage was to select more representative and discriminative features through the multivariant and univariant approach, respectively, after handling the imbalanced dataset. A machine learning predictive model was then constructed based on the selected features to classify the MSI status of colorectal cancer patients. In the last stage, we emphasized the significance of the selected features by analyzing their correlations with clinical features and conducting case studies.

### Radiomic Feature Extraction

The first principal stage was extracting the high-quantitative imaging features from the input PET/CT VOIs. The specific radiomic feature extraction process was summarized in **Supplementary Material Figure S1**. Before the extraction of these high-quantitative features, we applied different preprocessing settings to PET and CT images, respectively, to adapt to different image characteristics of these two modalities. PET images were discretized using a fixed bin width (FBW) of 0.25 (23–25) after applying the SUV normalization based on the body weights and the injection dose of the patients. Furthermore, other common parameters were applied as suggested in (23, 24, 26) to ensure the reproducibility of this process. As for CT images, the feature computation was performed at resampled voxel dimensions of 2 × 2 × 2 mm^3^ (26) and a bin width of 25 Hounsfield units (27, 28). We also shifted 1,000 voxel arrays for CT images to prevent negative values from being squared and add 10 extra paddings for large sigma-valued LoG-filtered CT images. Besides, the standard parameters for CT images were applied as suggested in (29).

There were 2,492 quantitative features extracted from PET/CT VOIs in total, of which 1,246 features were from PET VOIs and 1,246 features were from CT VOIs, through the open-source PyRadiomics package (29). The feature extraction procedure is compliant with the Imaging biomarker standardization initiative (30). The quantitative imaging features extracted from PET and CT VOIs are categorized into four subgroups. (1) Shape features are used to describe the shape of the focused region of interest (ROI) and its corresponding geometry properties such as volume, maximum surface. (2) First-order statistics features are used to describe the distribution of individual voxel values without considering the spatial relationships, such as the maximum, minimum value of the voxel intensities on the images (31). (3) Texture features are used to describe the statistical inter-relationships between neighboring voxels (32), which provide a spatial arrangement, such as gray-level co-occurrence matrix (GLCM), gray-level size zone matrix (GLRLM). (4) Higher-order statistics features are obtained through the statistical methods after applying filters or mathematical transforms to the images, which aims to identify the repetitive or non-repetitive patterns, or highlighting the details, such as wavelet transform and Laplacian of Gaussian (LoG) that can extract areas with increasingly coarse texture patterns (31).

### Feature Selection

The second stage started with handling the imbalanced training dataset and then followed by selecting representative and discriminative features through the multivariant and univariant approach sequentially. The entire 2010–2018 period CRC patients (n = 154) were stratified randomly split into training and independent validation cohorts according to the ratio of 6:4, which defines the study as a type 2a in the TRIPOD statement (33). The 19 additional 2018–2020 period patients with CRC were further used to enlarge the independent validation cohorts.

**Figure 2:**
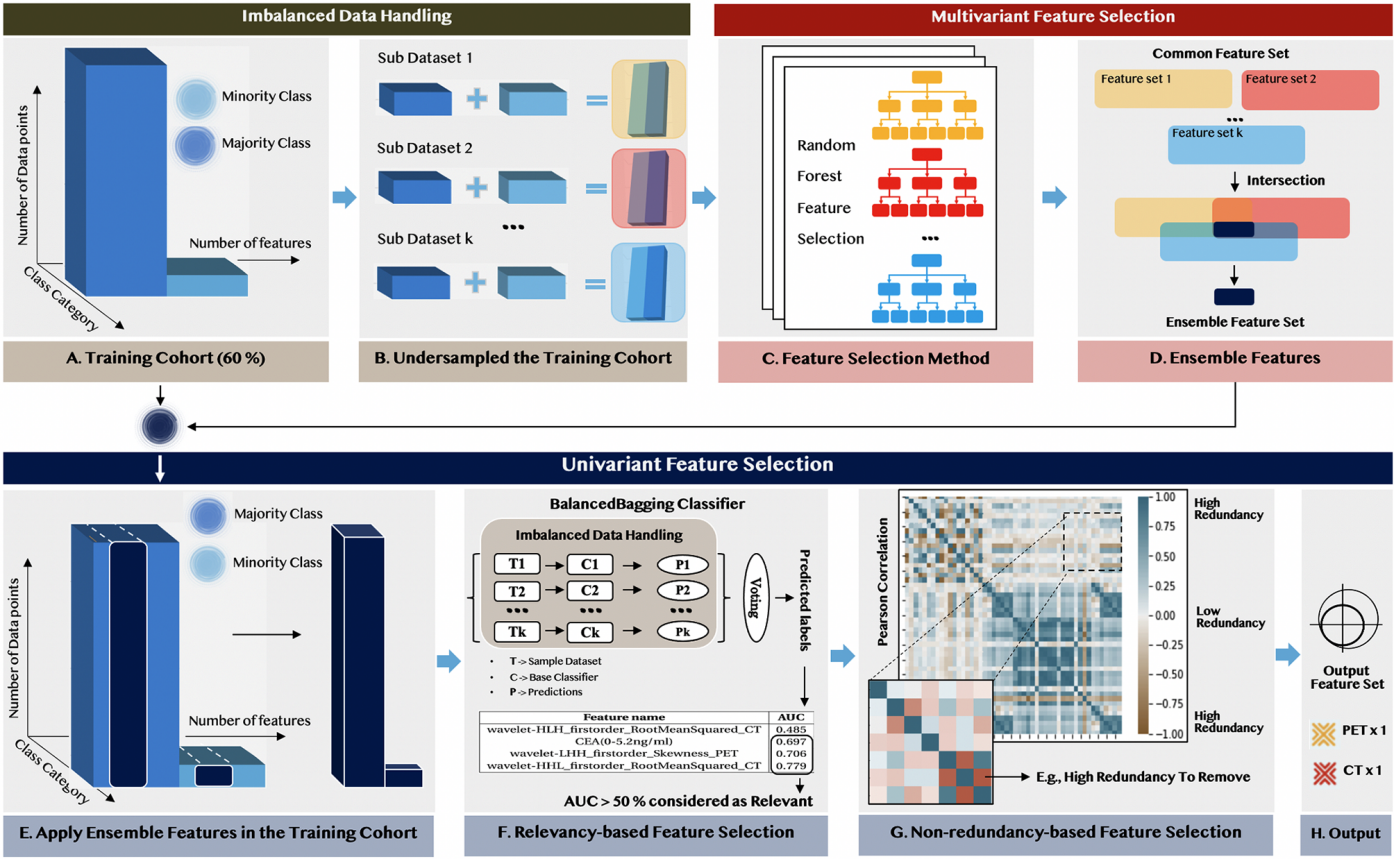
The flowchart of feature selection for selecting representative non-redundant and relevant features, as well as handling imbalanced data.

As illustrated in **Figure 2**, firstly, we applied the random under-sampling method (34) for handling the imbalanced dataset and obtained k sub-datasets with the same sample size of different target classes. This procedure ensured the selected features were equally significant to different prediction targets. Then, in multivariant feature selection, we exploited the ensemble paradigm (35, 36) to improve the robustness and the reproducibility of the selection process through the following steps: (1) We applied the random forest feature selection method (RF) with feature importance >0.01 to each sub-dataset and obtained k sub-feature sets. (2) We set a cut-off threshold to remove the contingency among these k sub-feature sets and the feature with less significance to both target classes. Only the feature that occurred among these feature sets multiple times (>5) was chosen and formed an ensemble feature set.

The univariant feature selection was deployed to remove the remaining redundant features from the internal properties and form the output feature set based on the ensemble feature set. Firstly, the relevancy-based analysis was conducted by evaluating the performance of each feature based on the BalancedBagging classifier incorporated with Adaboost as the base classifier. Then, the non-redundancy-based analysis was conducted based on the results from the Pearson correlation analysis among the selected radiomic features within the ensemble feature set.

### Modeling and Validation

The third stage was constructing the machine learning predicted model and validating its performance. The machine learning model was constructed based on the selected feature set using the BalancedBagging algorithm incorporated with Adaboost as the base classifier and trained on the training dataset. To validate the robustness and stability of the machine learning model, we only evaluated the model performance on the independent validation cohort due to the imbalanced nature of the dataset. The performance of the model was primarily evaluated by the AUC, while other metrics, including the sensitivity, specificity, positive predicted value (PPV), and negative predicted value (NPV), were used to detect the bias in the model.

### Statistical Analysis

Statistical analysis included result interpretation of the machine learning model and correlation analysis of selected radiomic with clinical features. To aid feature interpretations in both feature analysis and case studies, the Local Interpretable Model-Agnostic Explanations model (LIME) (37) was applied using its derived weight coefficients. It explains the contribution of each selected feature, thus gaining insights into the prediction model and assisting clinicians with trustworthy decisions. The LIME model assigned the higher weight coefficients to the features that the prediction results were more sensitive to through observing the changes of the results after eliminating several interpretable components.

To evaluate the correlation between the selected radiomic and clinical features, we applied the Pearson correlation method to measure the association between two continuous variables, while the Point-Biserial correlation was used for the measurement between one continuous variable and one categorical variable. All statistical analyses were implemented using the scikit-learn (sklearn) package (38) under Python version 3.6.4, and a two-sided P-value < 0.05 was considered statistically significant.

## Results

### Demographics of Patients

A total of 173 patients were finally enrolled in the study, of whom 160 were with MSI-L or MSS status and 13 were with MSI-H status. The middle age of the population was 61, ranging from 24 to 85 years old. According to the pathological results of the patients, about 38% of patients had extramural vascular invasion or perineural invasion. Besides, almost half of the population had a higher carcinoembryonic antigen (CEA) than the normal level (5.2 ng/ml). The clinicopathologic characteristics of the patients (n = 173) are summarized in **Table 1**, and the P-value was derived from the univariable correlation analysis between each characteristic and MSI status.

**Table 1:** Demographic and clinical characteristics of study subjects.

Characteristics		Total Population (n = 173)	MSI-H (n = 13)	MSS (n = 160)	P-value
Age, median (range)		61 (24~85)	61 (32~70)	61 (24~85)	0.454
Gender, n (%)		173	13	160	0.157
Stage, n (%)	Male	99 (57.2)	5 (38.5)	94 (58.8)	
	Female	74 (42.8)	8 (61.5)	66 (41.2)	**<0.001**
	I	11 (6.4)	3 (23.1)	8 (5.0)	
	II	46 (26.6)	7 (53.8)	39 (24.4)	
	III	65 (37.6)	3 (23.1)	62 (38.8)	
	IV	51 (29.4)	0 (0.0)	51 (31.8)	
CEA (ng/ml), n (%)					0.286
	≥5.2ng	83 (48.0)	11 (84.6)	72 (45.0)	
	<5.2ng	90 (52.0)	2 (15.4)	88 (55.0)	
Location, n (%)					**0.022**
	Ascending	18 (10.4)	2 (15.4)	16 (10.0)	
	Descending	21 (12.1)	3 (23.1)	18 (11.2)	
	Ileocecum	12 (6.9)	3 (23.1)	9 (5.6)	
	Rectum	63 (36.4)	4 (30.7)	59 (36.9)	
	Sigmoid	48 (27.8)	1 (7.7)	47 (29.4)	
	Transverse	11 (6.4)	0 (0.0)	11 (6.9)	
Extramural vascular invasion, n (%)					0.189
	+	55 (31.8)	2 (15.4)	53 (33.1)	
	−	118 (68.2)	11 (84.6)	107 (66.9)	
Perineural invasion, n (%)					**0.034**
	+	75 (43.4)	2 (15.4)	73 (45.6)	
	−	98 (56.6)	11 (84.6)	87 (54.4)	
40% MTV, mean (std)		20.64 (16.85)	25.75 (15.66)	20.22 (16.88)	0.258
SUVmax, mean (std)		14.65 (5.84)	15.25 (6.21)	14.60 (5.81)	0.699
SUVmean, mean (std)		8.80 (3.40)	8.99 (3.35)	8.79 (3.40)	0.839
TLG, mean (std)		188.74 (188.21)	254.79 (201.13)	183.37 (186.09)	0.190
Lymph nodes, mean (std)		2.60 (3.49)	0.38 (0.84)	2.78 (3.56)	**0.017**

The bold value means the value is smaller than 0.05 (< 0.05), and the corresponding feature is statistical correlated to the MSI status.

### Results of Feature Selection

**Figure 3:**
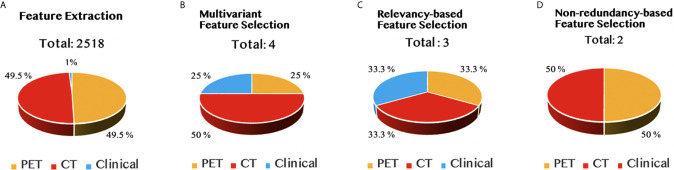
The results of feature selection in different stages, namely, **(A)** feature extraction, **(B)** multivariant feature selection, **(C)** relevancy-based feature selection, and **(D)** non-redundancy-based feature selection stage.

The result for each step of feature selection was illustrated in **Figure 3**. The entire feature selection was applied on 2,518 features, including 2,492 radiomic features and 26 clinical features. There were four features selected during the multivariant feature selection process, including one clinical feature, one feature obtained from the PET images that applied the wavelet filter, and two features obtained from wavelet decomposed CT images. The output feature set was composed of one CT feature and one PET feature after conducting the relevancy-based analysis and non-redundancy-based analysis sequentially.

### Performance of the Radiomics Signature

Since the original dataset was imbalanced, conducting the cross-validation method on the training group would cause a significant increase in the imbalanced ratio of the dataset. Therefore, the machine learning model was evaluated only with the independent validation method. During this procedure, the model correctly predicted the MSI status for 63 patients with 58 TN and 5 TP and incorrectly predicted the MSI status for 19 patients (18 FN and 1 FP). The machine learning model achieved a successful diagnosis in 76.8% (63/82) of patients’ MSI status, and the AUC of the model reached 82.8% with only two selected radiomic features. Its sensitivity and specificity were 83.3 and 76.3%, respectively, which stood for that the model did not show a trend of bias. The detailed radiomics signature performance is shown in **Table 2**, while the detailed parameter settings for each step were summarized in **Supplementary Table S1** for replication studies.

**Table 2:** Evaluation results with different output feature sets on independent validation dataset.

Feature	Accuracy	AUC	Sensitivity	Specificity
CEA (0–5.2 ng/ml)	0.610	0.692	0.5	0.618
CT feature only	**0.817**	**0.832**	0.5	**0.842**
PET feature only	0.439	0.684	0.667	0.421
**CT + PET**	0.768	0.828	**0.833**	0.763
**CEA + CT + PET**	0.744	0.803	0.667	0.750

The bold feature value represented the combined radiomic features that achieved high prediction accuracy for both target classes, while the bold numerical value represented the highest value of each column.

### Feature Analysis and Interpretation

There were two features identified by the sequentially combined multivariant and univariant feature selection process, namele, one PET feature (wavelet-LHH_firstorder_Skewness_PET) and one CT feature (wavelet-HHL_firstorder_RootMeanSquared_ CT). The details of these two selected features are shown in **Table 3**. It was relatively straightforward to observe that the two selected features were all from the wavelet decomposed images, which indicated that they were more predictive in comparison with the features obtained from the original images. The CT feature (HHL) captured the image texture information with high-pass filters along the first two dimensions, then filtered along the z-dimension with a low-pass filter, while the PET feature (LHH) captured the texture information through filtering along the x-dimension with a low-pass filter, followed by filtering the last two dimensions with high-pass filters.

**Table 3:** The definitions for the features selected for the predictive model construction.

Feature name	Feature definition and meaning
	**Formula**
**wavelet-HHL_firstorder_RootMeanSquared_CT**	$F_{stat,rms}=\sqrt{\frac{\Sigma_{k=1}^{N_{v}}(X_{gl,k}^{2})}{N_{v}}}$
	where *N_v_* represents the number of voxels,*X_d_* represents the set of intensities of the *N_v_* voxels included in the ROI intensity mask, which could be denoted as $X_{gl}=\{X_{gl,1},X_{gl,2}\ldots X_{gl,N_{v}}\}.$ Root mean square is the square-root of the mean of all the squared intensity values, which is a measure of the magnitude of the image values.
	**Formula**
**wavelet-LHH_firstorder_Skewness_PET**	$F_{ih,skew}=\frac{\frac{1}{N_{v}}\Sigma_{k=1}^{N_{v}}{\left(X_{d,k}-\mu \right)}^{3}}{{\left(\frac{1}{N_{v}}\Sigma_{k=1}^{N_{v}}N_{v}{\left(X_{d,k}-\mu \right)}^{2}\right)}^{\frac{3}{2}}}$
	where *N_v_* represents the number of voxels, *X_d_* is the set of discretized intensities of the voxels in the ROI intensity mask, which could be denoted as $X_{d}=\{X_{d,1},X_{d,2},\ldots X_{d,N_{v}}\}$ and *μ* is the average discretized intensity of *N_v_* voxels in the ROI intensity mask. Skewness is a measure of the asymmetry of the distribution of values about its mean by applying a wavelet filter, and its value could be positive or negative depending on the position that its tail is elongated and the position that the mass of the distribution is concentrated.

**Figure 4:**
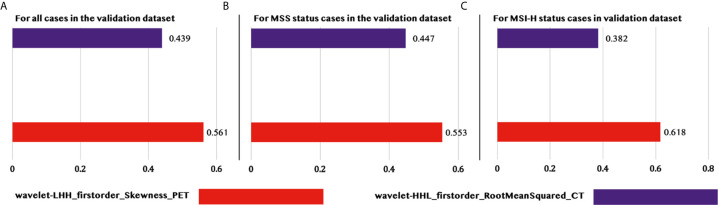
Feature importance for predicting MSI status of patients with CRCs in different situations. **(A)** Feature importance for predicting all the patients in the validation set. **(B)** Feature importance only for predicting the patients with MSS in the validation set. **(C)** Feature importance only for predicting the patients with MSI-H in the validation set.

Furthermore, the contribution of each feature to the model was illustrated in **Figure 4**, which indicated the normalized importance of each feature in three different situations, namely, (1) for all cases in the validation dataset, (2) for all MSS status cases in the validation dataset, and (3) for all MSI-H cases in the validation dataset. It was pretty evident that the PET_Skewness feature had a higher contribution than the CT_RootMeanSquared feature in all situations with contributions of 56.1, 55.3, and 61.8%, respectively, and it contributed most while predicting the MSI-H status in patients with CRC.

### Case Study

**Figure 5:**
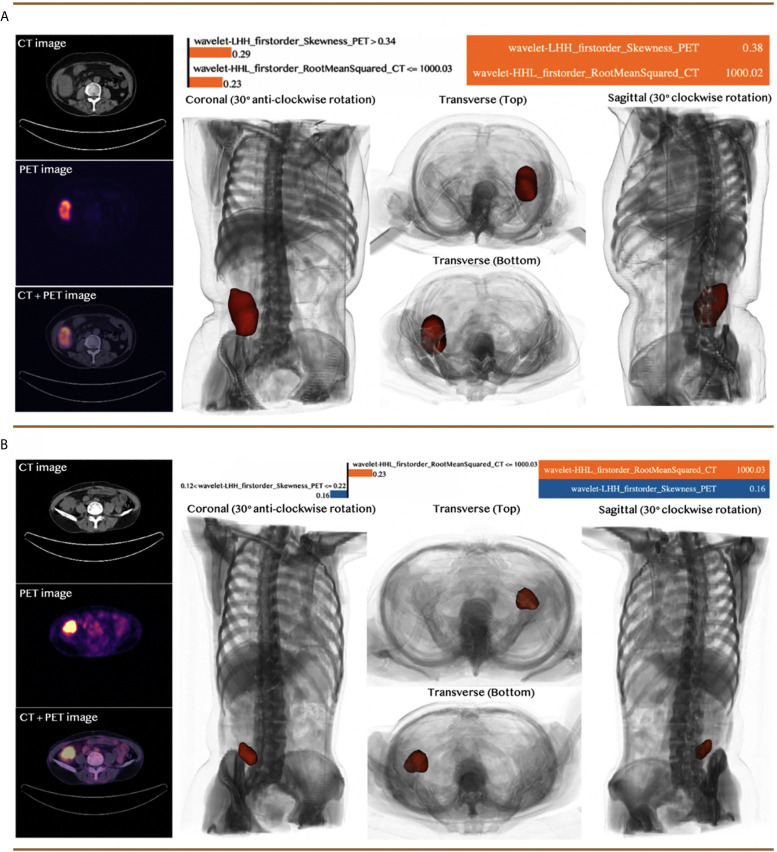
Case studies with two patients: **(A)** a patient with MSI-H status and **(B)** a patient with MSI-L or MSS status. Both patients were predicted correctly by the machine learning model. The top right section for each panel indicated the feature value of the corresponding case and the approximated feature weights interpreted by LIME. The bottom right section demonstrated the 3D model constructed based on the input CT and PET images from different viewpoints, while the red section represented the tumor of the patients.

We selected two CRC patients with different MSI statuses from the independent validation dataset to demonstrate the performance of the predictive model. The visual analysis and the machine learning model for the two selected cases were illustrated in **Figure 5**, including the 3D model of each patient constructed by the input PET/CT images, the value of the selected features, and the weight coefficients derived by the LIME model for each feature. The specific feature values for each case were indicated in the table on the top right corner of each panel in **Figure 5**, while the specific numbers near the bar chart were the weight coefficients applied in the prediction of these two typical clinical cases, whose linear combination with the corresponding feature values provided the prediction results. The value of the orange bar indicated the increase of the corresponding features was supporting the decision of MSI-H status, while the value of the blue bar was the opposite. The prediction results were determined by the side with a higher value. The model quantitatively combined the selected features with their diverse weight coefficients for the final prediction and correctly predicted for both cases.

### Correlation Analysis Between Selected Radiomic Features and Clinical Features

**Figure 6:**
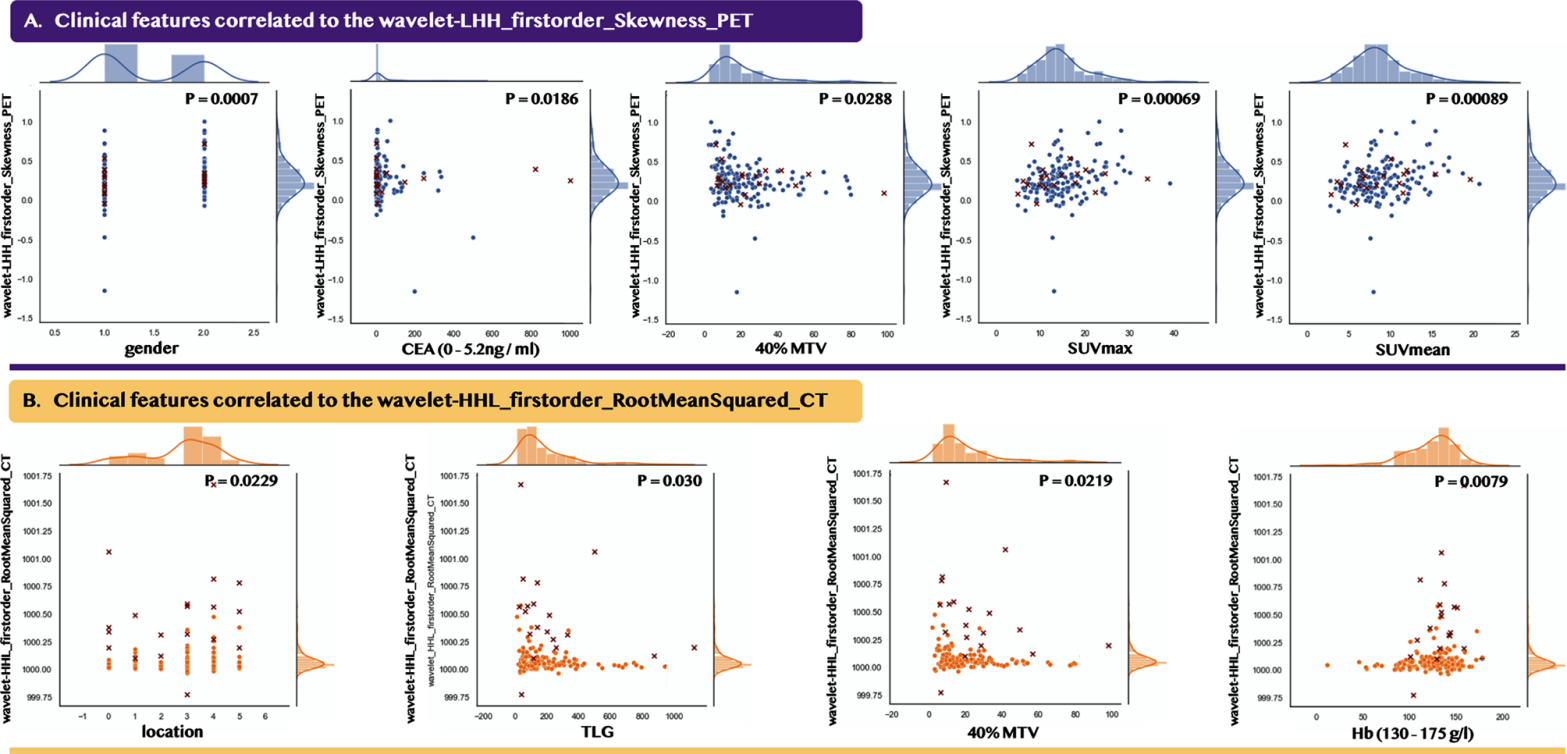
Data distribution between the selected radiomic features and their statistically correlated clinical features. Point mark and cross mark indicate the patients from the 2010–2018 and 2018–2020 periods, respectively. **(A)** indicates the PET feature, while **(B)** indicates the CT feature. For gender and location, we applied the Point-Biserial correlation method, while for the other clinical features, we applied the Pearson correlation method.

**Figure 6** indicates the selected two radiomic features were statistically correlated to nine clinical features. The blue figures show that the PET_Skewness feature correlated to five clinical features, while the yellow figures indicate the CT_RootMeanSquared feature was statistically correlated to the remaining four clinical features. Since most of the statistically correlated clinical features were medically defaulted to be effective in diagnosing microsatellite statuses such as location and CEA (0–5.2 ng/ml), it could well explain the low number of the selected features and the significance of the selected features.

## Discussion

This study aimed to investigate the value of preoperative ^18^F-FDG PET/CT radiomics signature with the machine learning model to predict MSI status in CRC patients. Our main findings were indicated as follows: (1) We provided an efficient, objective, and non-invasive mechanism by establishing and validating a radiomics predictive model (AUC 82.8%) using preoperative ^18^F-FDG PET/CT to identify MSI status in patients with CRC before surgery. (2) Two selected radiomic features were used for constructing the predictive model, and they were identified to be significantly correlated with multiple clinical predictive features that were previously proved to be associated with MSI status. Our radiomics model established a new method for testing MSI status, which assisted in predicting prognosis and choosing a proper treatment strategy in CRC patients.

We firstly investigated the characteristics of the patients with different MSI statuses in the dataset, with a primary focus on different CRC stages, and then compared with the other studies to point out the clinical significance of our study. In colorectal cancer, MSI status correlates with survival prognosis and the decision-making in adjuvant chemotherapy and immunotherapy. Therefore, it is crucial to identify the MSI status for individual patient to achieve survival benefit through a non-invasive method. The incidence of MSI-H in sporadic colorectal cancer was about 15% (39), while only 7.5% (13/173) of patients were with MSI-H in our study, which might be caused by more stage IV patients (29.4%, 51/173) enrolled. The previous report showed that below 5% of metastatic CRCs were with MSI-H in stage IV CRCs (5), while no patients were with MSI-H in 51 stage IV patients in our study. Consistent with the previous finding that MSI-H CRCs were more frequent in stage II (40), the rate of MSI-H in stage II CRCs (53.8%) was considerably higher compared to that in other stages. Most of the studies demonstrated that MSI-H CRCs were more commonly localized in the right colon (2). Moreover, in this study, more MSI-H lesions were located in the left colon, which could be caused by the large population of rectal cancer. Our results also revealed that less perineural invasion occurred in patients with MSI-H than ones with MSI-L or MSS, which was pointed out in a study on immune contextures of gastric cancer that a high level of CD3+ and CD8+ tumor-infiltrating lymphocytes (TILs) were associated with perineural invasion and MSI-H (41).

Our proposed radiomic model based on ^18^F-FDG PET/CT is non-invasive and objective when compared with two conventional invasive methods recommended for identifying MSI status, namely, (1) testing the loss of four MMR proteins expression by IHC and (2) detecting the repetitiveness of DNA sequence by PCR methods. Immunohistochemistry was a low-cost technique and was routinely used in the department of pathology. However, the sensitivity of IHC would be influenced and reduced by the fixation of tissue samples and neoadjuvant chemoradiation. While for the PCR method, a panel of five microsatellite repeats, called Bethesda panel, was widely used for detecting MSI status. MSI high was defined as the detection of at least two markers while comparing marker length between normal and tumor tissue. Besides, poor sensitivity of the PCR method could be caused by tumor samples with low levels of tumor cells, which were achieved by biopsies, or from mucinous tumors and tumors after neoadjuvant chemoradiation (42). Since ^18^F-FDG PET/CT-based radiomics was not influenced by the treatment approach, routine baseline imaging could provide additional diagnostic information for the identification of MSI status through a non-invasive approach.

Since ^18^F-FDG PET/CT reflected anatomic morphology and glucose metabolism, which contained lots of information about prognosis and treatment response, we primarily conducted a univariant association analysis among metabolic PET parameters (SUVmax, SUVmean, MTV, and TLG) for different MSI statuses, and it indicated no significant difference between MSI-H and MSI-L or MSS (p = 0.374, 0.389, 0.102, 0.141). However, gastric cancer research exhibited that MSI-H tumors caused higher SUVmax on ^18^F-FDG PET/CT imaging (43). The difference between that research and ours was that gastric cancer with MSI-H tended to be larger-sized and histologically heterogeneous, which was different from colorectal cancer (44). Besides, more early-stage patients were enrolled in that research, while there were more stage III and IV patients enrolled in our study. Few reports about the association of PET/CT and MSI status illustrated the importance and necessity of our study. The present results indicated that the conventional metabolic PET parameters were not a significant clinical predictor for MSI status.

After the excavation of image datum, our study analyzed all stage I–IV patients, filtered out only two features, one from PET (LHH) and one from CT (HHL), and achieved a successful diagnostic efficiency using the machine learning model. Its AUC, accuracy, sensitivity, and specificity were 82.8, 76.8, 83.3, and 76.3%, respectively. Pernicka (45) proposed a radiomics approach that achieved an AUC of 0.792 through a combination of clinical and radiomic features, while Fan et al. (46) combined six radiomic features and 11 clinical features to establish a model to predict MSI status in stage II CRC and achieved the AUC, accuracy, sensitivity, and specificity of 0.752, 0.765, 0.663, 0.842. These results showed the value of radiomic features of preoperative imaging of primary tumors for predicting microsatellite status.

Even though the radiomics-based model developed by Pernicka (45) achieved a relatively high AUC (0.792), it showed an unsatisfactory specificity (0.316) on the independent validation cohorts, which might be caused by the high number of features (42 features out of 198 patients) employed within the model, including 40 intensity-based radiomic features, and two clinical features (age and tumor location). According to the theory of Chalkidou et al. (47), the number of employed features was expected no more than 15% of the patients involved in the study to reduce the false detection rates and prevent the model from overfitting. As compared with 17 features employed in Fan et al.’s study, our PET/CT study selected only two radiomic features and achieved comparable diagnostic results. Among the six selected radiomic features in their study (46), consisting of one kurtosis feature (first-order statistical feature class) and five Gabor filter features (higher-order statistical features), five shared the same features class as the two selected features (wavelet filter) in our study. According to the feature importance ranking demonstrated within their study (46), the five higher-order statistical features contributed more to the prediction of MSI-H status. In addition, to better support the clinical decision, our framework integrated the interpretation capacity through (1) gaining insights into each prediction based on the LIME model (37) to obtain a better understanding of the radiomics-based model for MSI status identification, and (2) conducting the statistical analysis with clinical features and histological characteristics, including tumor stage, perineural invasion, and extramural vascular invasion.

We emphasized the reliability and the significance of our selected features through statistical correlation analysis with the clinical features. According to the results shown in **Figure 6**, the Skewness_PET feature was correlated to five predictive clinical features, namely, gender, CEA, and three PET metabolic parameters (MTV, SUVmax, and SUVmean); while the RootMeanSquare_CT feature was correlated to four predictive clinical features, namely, MTV, TLG, Hb, and location. In previous researches, colorectal cancer with MSI was associated with female gender (2, 48), abnormal CEA, and right-side tumor location. Our results showed that were more female patients with MSI than male patients. Female patients were more likely to have MSI, which was found in colorectal cancer and gastric cancer (49). Carcinoembryonic antigen (CEA) is an important biomarker in predicting colorectal cancer progression. A prospective study in Indian populations demonstrated that a strong positive correlation was found between CEA and MSI status (50), and both could predict the prognosis of CRC. Our study also found that more patients with elevated CEA showed MSI-H (**Table 1**). Although the single PET metabolic parameter could not predict MSI status precisely, we observed that our selected radiomic features were correlated to several PET metabolic parameters. It was obvious that the filtered features were valuable in clinical prediction. Through the integration of these clinical features, including the PET metabolic parameters, our machine learning model realized the possibility of predicting MSI status by simple two features from PET and CT, respectively.

## Conclusion

In conclusion, we established a radiomics predictive model that incorporated ^18^F-FDG PET/CT radiomic signatures and clinical features to provide a non-invasive and objective mechanism to identify MSI status in patients with CRC preoperatively. The two selected PET/CT radiomic features, each significantly correlated with multiple clinical features, achieved high diagnostic performance, which potentially facilitated the individualized treatment and prognosis prediction.

## Data Availability Statement

The original contributions presented in the study are included in the article/**Supplementary Material**. Further inquiries can be directed to the corresponding authors.

## Ethics Statement

The studies involving human participants were reviewed and approved by Ethics Committee of Fudan University Shanghai Cancer Center (No. 1909207-14-1910). The patients/participants provided their written informed consent to participate in this study.

## Author Contributions

All authors were involved with the conception and design, manuscript writing, and final approval of the manuscript.

## Funding

This work was partially supported by the National Natural Science Foundation of China (81771861, 81971648), Shanghai Scientific and Technological Innovation Program (No. 18410711200, 19142202100), and National Key Research and Development Project (No. SQ2019YFC160090/05).

## Conflict of Interest

The authors declare that the research was conducted in the absence of any commercial or financial relationships that could be construed as a potential conflict of interest.

## Supplementary Material

Supplementary Figure S1 The detailed process of radiomic feature extraction.

Supplementary Table S1 The related parameter settings for replication studies.

## Abbreviations

AUC, area under the curve; CEA, carcinoembryonic antigen; CRC, colorectal cancer; CT, computed tomography; CTLA-4, cytotoxic T lymphocyte-associated antigen 4; dMMR, mismatch repair-deficient; FBW, fixed bin width; FDA, Food and Drug Administration; FDG, fluorodeoxyglucose; FN, false negative; FP, false positive; GLCM, gray-level co-occurrence matrix; GLRLM, gray-level size zone matrix; Hb, hemoglobin; HHL, high, high, and low frequency; HNPCC, hereditary non-polyposis colorectal cancer; IHC, immunohistochemical staining; LHH, low, low, and high frequency; LIME, Local Interpretable Model-Agnostic Explanations model; LoG, Laplacian of Gaussian; LS, lynch syndrome; MMR, mismatch repair; MSI, microsatellite instability; MSI-H, microsatellite instability-high; MSI-L, microsatellite instability-low; MSS, microsatellite stable; MTV, metabolic volume; NGS, next-generation sequencing; NPV, negative predicted value; PCR, polymerase chain reaction; PD-1, programmed death-1; PD-L1, programmed death-ligand 1; PET, positron emission tomography; pMMR, proficient mismatch repair; PPV, positive predicted value; RF, random forest; ROI, region of interest; sklearn, scikit-learn; SUV, standardized uptake value; SUVmax, the maximum standardized uptake value; SUVmean, the mean standardized uptake value; T2WI, T2 weighted images; Th1, T helper 1; TIL, tumor-infiltrating lymphocyte; TLG, total lesion glycolysis; TN, true negative; TP, true positive; TRIPOD, transparent reporting of a multivariable prediction model for individual prognosis or diagnosis; VOI, volume of interest.
