# Supplementary figures and images for: Quantitative Prediction of Microsatellite Instability in Colorectal Cancer With Preoperative PET/CT-Based Radiomics
Source: Front Oncol. 2021 Jul 22;11:702055. doi: 10.3389/fonc.2021.702055 (PMC8339969; doi:10.3389/fonc.2021.702055)

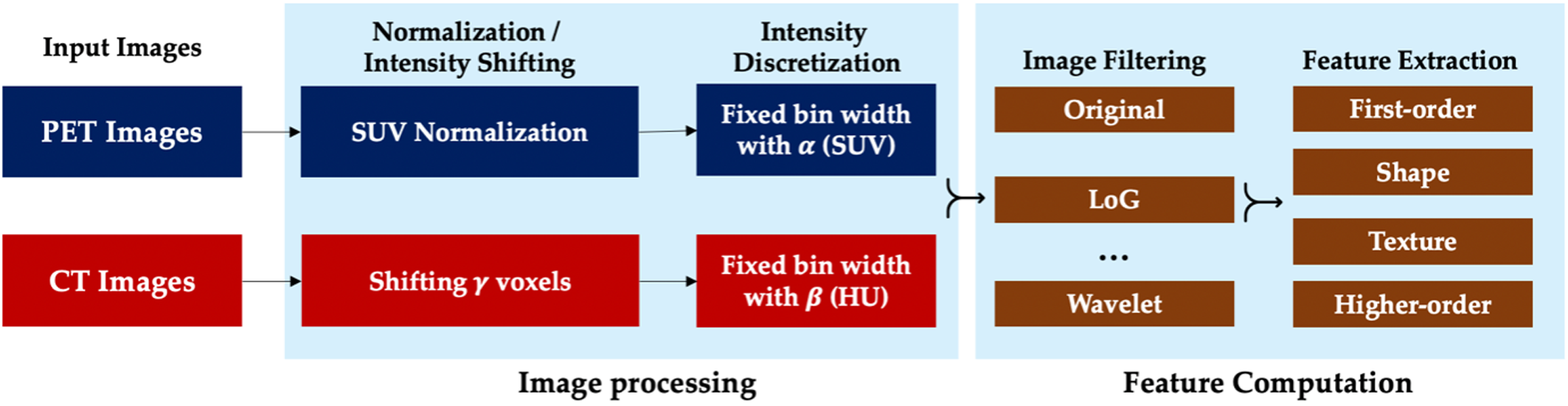

Supplement: Supplementary Figure S1 — The detailed process of radiomic feature extraction. [file Image_1.jpeg]
