# Supplementary material for: Quantitative Prediction of Microsatellite Instability in Colorectal Cancer With Preoperative PET/CT-Based Radiomics
Source: Front Oncol. 2021 Jul 22;11:702055. doi: 10.3389/fonc.2021.702055 (PMC8339969; doi:10.3389/fonc.2021.702055)
Supplement: Supplementary Table S1 — The related parameter settings for replication studies. [file Table_1.docx]

**Table S1** The related parameter settings for replication studies.

| **PET/CT Acquisition Parameter Settings** | resolution  voxel size (mm^3^) | | **PET**  168 x 168 x 172  4.06 x 4.06 x 5 | **CT**  512 x 512 x 172  1.37 x 1.37 x 5 |
| --- | --- | --- | --- | --- |
| **Feature Extraction Parameter Settings** |  | **PET** | | **CT** |
|  | voxelArrayShift | 0 (default) | | 1000 |
|  | resampledPixelSpacing (mm^3^) | 1 x 1 x 1 | | 2 x 2 x 2 |
|  | FBW | 0.25 (SUV) | | 25 (HU) |
|  | padDistance | 5 (default) | | 10 |
|  | geometryTolerance | 0.001 | | 1e-16 (default) |
|  | Interpolator | sitkBSpline | | sitkBSpline |
| **Feature Selection Parameter Settings** | **Multivariant** |  | |  |
|  |  | # of sub datasets (k) | | 30 |
|  |  | classifier | | random forest |
|  |  | n_estimators | | 100 |
|  |  | feature importance | | > 0.01 |
|  |  | intersection threshold | | 5 |
|  | **Relevancy-based (univariant)** |  | |  |
|  |  | classifier | | BalancedBagging |
|  |  | base classifier | | Adaboost |
|  |  | n_estimators | | 11 |
|  |  | boostrap | | True |
|  | **Non-redundancy based (univariant)** |  | |  |
|  |  | correlation method | | Pearson correlation |
|  |  | P-value | | <0.05 |
|  |  |  | |  |
| **Model Construction Parameter Settings** |  | classifier  base classifier  n_estimators | | BalancedBagging  Adaboost  11 |
| **Statistical Analysis Parameter Settings** | **LIME model** | classifier  base classifier  algorithm  boostrap  n_estimators  random-state  oob_score  sampling strategy  verbose  warm_start | | BalancedBagging  Adaboost  SAMME.R  True  11  0  False  auto  0  False |
|  | **Correlation Analysis** | Pearson Correlation method and Point-Biserial Correlation method | | |
| **Original Dataset Split Ratio** | Training Cohort: Independent Validation Cohort | | | 6:4 |
| **Software Information** |  | Python  PyRadiomics  Sklearn | | 3.6.4  2.0.1  0.23.2 |
